# Potential Sources, Pollution, and Ecological Risk Assessment of Potentially Toxic Elements in Surface Soils on the North-Eastern Margin of the Tibetan Plateau

**DOI:** 10.3390/toxics10070368

**Published:** 2022-07-03

**Authors:** Yujun Ma, Qiugui Wang, Weigang Su, Guangchao Cao, Guoyan Fu, Wen Du

**Affiliations:** 1 College of Geographic Science, Qinghai Normal University, Xining 810008, China; 2016130@qhnu.edu.cn; 2 Key Laboratory of Tibetan Plateau Land Surface Processes and Ecological Conservation (Ministry of Education), Qinghai Normal University, Xining 810008, China; 3 Qinghai Province Key Laboratory of Physical Geography and Environmental Process, Qinghai Normal University, Xining 810008, China; 4 Key Laboratory for Water Quality and Conservation of the Pearl River Delta, Ministry of Education, School of Environmental Science and Engineering, Guangzhou University, Guangzhou 510006, China; wangqiugui@gzhu.edu.cn; 5 Qinghai Earthquake Agency, Xining 810001, China; yangyuwan_su@163.com; 6 Key Laboratory of Comprehensive and Highly Efficient Utilization of Salt Lake Resources, Chinese Academy of Sciences and Qinghai Provincial Key Laboratory of Geology and Environment of Salt Lakes, Qinghai Institute of Salt Lakes, Chinese Academy of Sciences, Xining 810016, China; 7 Zhongyuan Institute of Science and Technology, Zhengzhou 450000, China; fgy8023@126.com; 8 School of Mathematics and Statistics, Qinghai Normal University, Xining 810008, China; 20201089@qhnu.edu.cn

**Keywords:** potentially toxic element, northeastern margin of the Qinghai–Tibet plateau, different landscape areas, soil, pollution, risk assessment

## Abstract

Due to increased levels of human activity, various pollutants are frequently detected on the Tibetan Plateau, where the environment is extremely fragile and sensitive. Therefore, this study investigated the sources, pollution, and ecological risks of soil potentially toxic elements (PTEs) in different landscape areas within the Qaidam Basin in the northeastern part of the Qinghai–Tibet Plateau. The contents of seven PTEs (Cd, Cu, Pb, Zn, As, Cr, and Ni) in 32 topsoil samples (0–2 cm) were analyzed in different regions of the Qaidam Basin. The concentrations of As, Cd, Cr, Cu, Ni, Pb, and Zn were 10.4–29.9 mg/kg, 0.08–4.45 mg/kg, 19–66 mg/kg, 8.2–40 mg/kg, 11.7–30.8 mg/kg, 11.1–31.2 mg/kg, and 32–213 mg/kg, respectively. The correlation between Pb and Cd in unpopulated areas was 0.896 (*p* < 0.01). The correlations among Pb, Cd, and Zn in agricultural areas, among As, Cd, Cr, and Zn in saline lake areas, and among As, Cd, Cr, Cu, Ni, Pb, and Zn in residential areas were all greater than 0.65 (*p* < 0.05). The principal component analysis results showed that Pb and Cd in unpopulated areas, Pb, Cd, and Zn in agricultural areas, As, Cd, Cr, Zn, and Pb in saline lake areas, and As, Cd, Cr, Cu, Ni, Pb, and Zn in residential areas were affected by human activities (significant factor >0.70). Based on the geological accumulation index and single-factor pollution index results, the maximum Cd values were found to be 4.93 and 45.88, respectively; Cd was thus the most serious PTE pollutant. The comprehensive pollution index of Nemero showed that moderately and severely polluted areas accounted for 18.89% and 18.46% of the total area, respectively. The results of the potential risk index showed that very strong and strong ecological risk points together accounted for 18.8% of the total points. The spatial variations in PTE pollution and the potential ecological risk index had similar patterns; both increased from the unpopulated areas in the northeastern Qaidam Basin to Golmud city in the south-western Qaidam Basin. These results indicate that human activities negatively impacted the soil ecological environment in the Qaidam Basin during the rapid development of the economy and urbanization and that these negative impacts tended to spread to unpopulated areas. Therefore, it is necessary to emphasize the significant impacts of human activities on environmental quality and formulate preventive measures to reduce PTE pollution in the Qinghai–Tibet Plateau.

## 1. Introduction

The increasing concentrations of PTEs caused by human activities have led to a series of serious environmental problems, such as air pollution, water pollution, and soil pollution [1,2,3]. Soils are important sinks of nutrients and pollutants which play a crucial role in ecological stability and security [4]. However, soil pollution has become a serious obstacle to regional development and human health in recent decades [5,6]. As a major soil pollutant type, PTEs have become important pollutants in soils around the world [7] because of the rapid economic development and the development of metallurgical technology, the amounts of PTEs emitted to the atmosphere have increased locally and globally [8] and the PTEs pollution has become increasingly serious worldwide and has attracted global attention due to the associated environmental toxicity, enrichment, and persistence [9]. The presence of PTEs in soils originates from natural and human activities [10], and the anthropogenic origins mainly include mineral mining [11], smelting [12], fossil fuel combustion [13], and agricultural activities [14]. With the occurrence of industrialization and urbanization processes after the industrial revolution, the concentrations of PTEs in soils are increasing regionally and globally [8]. Even in remote and sparsely populated areas, the accumulation of PTEs in soils cannot be ignored [15,16,17]. PTEs can be adsorbed on the particulate matter to undergo long-distance transportation through the atmosphere [8] and finally deposited into soils in remote areas, ultimately leading to the enrichment of PTEs in these soils [16]. Previous studies have shown that soils in the Third Pole [4], Antarctica [18], the Arctic region [19], and high-latitude Siberia [20] were enriched with PTEs.

Considered the “third pole of the world,” the Tibetan Plateau is one of the most remote and isolated regions in the world [17]. Due to its low urbanization and industrialization levels [15], most of the regional ecosystems on the Tibetan Plateau are well preserved. Its unique topography, fragile ecosystems, and special monsoon circulation [14] make it highly sensitive to external influences. Even small-scale human activities may lead to significant environmental changes [21]. Previous studies have shown that the highest Pb, Zn, Cr, and Cd concentrations in the soils on the Qinghai–Tibet Plateau can reach 1075.69 mg/kg, 1474 mg/kg, 153.61 mg/kg, and 11.56 mg/kg, respectively [22]. Therefore, soil PTE pollution on the Qinghai–Tibet Plateau should not be ignored. Existing studies have mainly focused on investigating PTEs in Qingha-Tibet soils [4,23,24], glacial soils [25], railways [22], highways [15] and the litter catchment [26]. And found that the cryosphere (glaciers, permafrost, ice, and snow) was the essential source of PTEs during climate warming [27]. However, regrettably, these studies still make it hard to understand the source of PTEs from located human activities, such as agricultural activities, mining activities, and urbanization in a different region of Qinghai–Tibet Plateau, and they did not adequately elucidate the contamination, ecological or health risks associated with PTEs in soils and the sources of PTEs in soils in different land-use type in Qinghai–Tibet Plateau.

The Qaidam Basin is located at the northeastern edge of the Qinghai–Tibet Plateau and is the region of the plateau with the largest population and the highest degree of industrialization [15]. Saline lakes and the Gobi Desert are typical landscapes in this area. With the increase in human activities, PTE pollution in the region is becoming increasingly serious. Previous studies have mainly focused on the distributions of PTEs in the soils along with expressway and railway areas in the northeastern region of this area [15,22]. However, research on the sources of soil PTEs regarding different human activities and their impacts on local ecosystems is lacking. Therefore, the purpose of this study was to investigate the distribution of soil PTEs under the influence of different human activities on the northeastern margin of the Qaidam Basin, the potential sources of the identified soil PTEs, and the impacts of these PTEs on the local environment and ecology. The results can provide a different perspective for soil pollution assessments and soil protection measures on the Qinghai–Tibet Plateau.

## 2. Materials and Methods

### 2.1. Research Area

The research area is located in the Qaidam Basin on the northeastern margin of the Qinghai–Tibet Plateau. The Qaidam Basin is a large inland discontinuous mountain basin with a frequently cold and warm climate and a dry-and-wet climate succession pattern. The basin is filled with Mesozoic–Cenozoic sediments derived from the surrounding mountains, and the sedimentary sequence is more than 16,000 m thick and lies on top of the pre-Mesozoic basement which comprises metamorphic and igneous rocks [28]. The formation of regional topography is closely related to the uplift of the Qinghai-Tibet Plateau, and the area is also sensitive to climate change across the whole Qinghai-Tibet Plateau. The basin has a plateau continental climate, with annual precipitation decreasing from 200 mm in the southeast to 15 mm in the northwest. The annual relative humidity is 30–40%, the annual average temperature is below 5 °C, and the absolute annual temperature difference can reach over 60 °C. The ecological environment of the study area is fragile, and small environmental changes can thus critically impact the local ecology.

### 2.2. Sample Collected and Chemical Analysis

**Figure 1:**
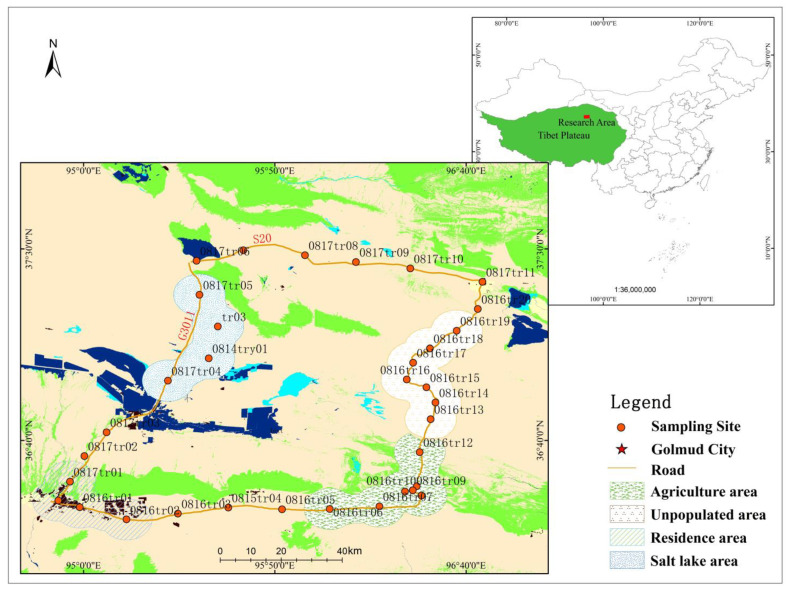
Sampling sites for soils in Qaidam Basin.

Samples were collected in August 2020 along a sampling route from G3011 and G109 to Naomuhong town, where the transect intersected with G315 to form a closed ring (Figure 1). From Nuomuhong to G315, economic (medlar) forests are widely distributed. A total of 32 surface soil samples were collected, including 7 samples in the agricultural areas, 7 samples in the unmanned areas, 4 samples in the residential areas, 4 samples in the salt lake areas, anfigurefid others collected on the highway roads. The samples were collected at a 10-cm × 10-cm × 2-cm volume in the surface layer [29], the sample was 2 cm in depth [29], and the average weight of collected samples was approximately 8 g. After being collected, the samples were placed into a polyethylene ziplocked bag, numbered, sealed, weighed, and taken back to the laboratory. Then, they were placed in a constant-temperature oven and dried at 60 °C until they reached a constant weight. Stones, roots, and other particles were removed from the samples by passing them through a 100-mesh sieve. Finally, the screened samples were packed into polyethylene ziplocked bags and stored after being numbered.

The method described by Kuklová et al. [30] was used to pre-treat the samples before the elemental content determination. A given weight of a mixed sediment sample (~125 mg) was placed in a 50 mL Teflon beaker. After that, the sediment was digested by microwaves with a volumetric mixture of concentrated HNO_3_ + HCl + HF + HClO_4_ at a ratio of 1:1:1:1 until the sample was completely dissolved. The residue was then transferred to a Teflon beaker, placed on a heating plate at approximately 200 °C until the sample was dry, and then diluted with deionized water to 25 mL. The measurement of the PTEs in each sample was determined by inductively coupled plasma atomic emission spectroscopy (ICP-AES; Agilent VISTA, La Jolla, CA, USA) and inductively coupled plasma mass spectrometry (ICP–MS; Agilent 7700X, La Jolla, CA, USA). The PTEs measurements were quality controlled using blank samples, repeated samples, and separately determined reference substances (OREAS 90 and GBM908-10). The relative deviation and relative precision control error were both less than 10%. The analytical accuracy of the samples was reported at a confidence level above 95%. Elemental determination was performed in the Mineral Analysis Laboratory of Aoshi Analytical Testing in Guangzhou, China.

### 2.3. Evaluating the Pollution and Ecological Risks of PTEs in Soils

This study used the geological accumulation index (*I_geo_*), single factor pollution index (*P_i_*), and Nemerow pollution index (*NPI*) to evaluate the pollution of PTEs in the topsoil on the northeastern Qinghai–Tibet Plateau. The background concentrations of PTEs in soils used to calculate *I_geo_*, *P_i_*, and *NPI* were established by referring to MEPC (1990) [31]. The *I_geo_*, *P_i_,* and *NPI* classification schemes are shown in Table 1.

*I_geo_* was employed to assess the PTE pollution level in sediments. *I_geo_* can be calculated as follows [32]:

$$I_{geo}=\log_{2}\frac{C_{s}^{i}}{1.5\times C_{b}}$$

where *C_s_^i^* is the concentration of PTE *i* in the sample, *C_b_* is the background concentration value, and the background values of As, Cd, Cr, Cu, Ni, Pb, and Zn are 11.2 mg/kg, 0.097 mg/kg, 61 mg/kg, 22.6 mg/kg, 26.9 mg/kg, 26 mg/kg, and 74.2 mg/kg, respectively [31].

*P_i_* can be used to evaluate changes in the amounts of individual PTE elements in the soil. The calculation formula of the single-factor pollution index method is as follows [33]:

$$Pi=\frac{C_{s}^{i}}{C_{b}}$$

where *P_i_* is the pollution index of PTE element i in soil. The pollution grade standards are shown in Table 1.

*NPI* is used to evaluate the overall situation of PTEs in soils. This index considers not only the effects of PTEs with high concentrations on the environment but also the impact of each PTE on the environmental quality by analyzing its average value. *NPI* is calculated by the following equation [34]:

$$NPI=\sqrt{\frac{(max(Pi){)}^{2}+(avg(Pi){)}^{2}}{2}}\;$$

where *NPI* is the comprehensive pollution index of the sampling point; *P_i_* is the single-factor index evaluation value of the PTE *i*; *max (P_i_)* is the maximum value of *P_i_*, and *avg (P_i_)* is the average value of *P_i_*. The pollution classification scheme is shown in Table 1.

PTEs exert potential ecological risks to soil systems. The potential ecological risk index (RI) represents the sensitivity of a biological community to pollutants and illustrates the resulting potential ecological risk [33]. The equation used to calculate RI is as follows:

$$RI=\overset{n}{\underset{i=1}{\sum }}E_{r}^{i}=\overset{n}{\underset{i=1}{\sum }}T_{r}^{i}\times P_{r}^{i}\;$$

where *RI* is the potential ecological risk index; *E^i^_r_* is the potential ecological risk coefficient of a single PTE, and *T_r_^i^* is the toxicity coefficient of a single PTE. The toxicity coefficients of PTEs are as follows: As = 10, Cu = Ni = Pb = 5, Zn = 1, Cr = 2, and Cd = 30. Pir is a single-factor index of the measured values of the PTEs. The RI classification scheme is shown in Table 1.

## 3. Results and Discussion

### 3.1. Spatial Distributions of PTE Concentrations

The variation of PTEs were shown in Table 2. The mean values were 18.2 ± 10.3 mg/kg, 0.02 ± 0.01 mg/kg, 0.38 ± 0.74 mg/kg, 39.6 ± 10.4 mg/kg, 19.1 ± 5.28 mg/kg, 34.5 ± 41.1 mg/kg, and 75.8 ± 36.5 mg/kg for As, Cd, Cr, Cu, Ni, Pb, and Zn, respectively. The variation coefficients of the seven PTEs measured in the topsoil in the study area were ranked in the following order: Cd (194.11%) > Pb (119.04%) > As (56.34%) > Zn (48.16%) > Cu (40.11%) > Ni (27.68%) > Cr (26.31%). The variation coefficients of Cd (194.11%) and Pb (119.04%) were large, suggesting that the Cd and Pb contents varied greatly in the study area. However, the coefficients of variation in Cr (26.31%) and Ni (27.68%) were less than 35%, indicating that the regional distributions of Cr and Ni are relatively stable and may be less disturbed by human activities than those of other PTEs [35]. The average values of Cr, Cu, and Ni derived in this study were lower than those calculated by predecessors (58.46 mg/kg, 20.16 mg/kg, and 23.78 mg/kg, respectively; [36]), while the average values of Pb and Zn were higher than those reported by Yang et al. (2021) [36] for the surface soil of the Qaidam Basin (20.37 mg/kg and 57.2 mg/kg, respectively). The Cd, Cr, Cu, Ni, Pb, and Zn concentrations were lower than their average values of 0.68 mg/kg, 93.29 mg/kg, 40.74 mg/kg, 54.73 mg/kg, 72.49 mg/kg, and 145.64 mg/kg, respectively, in the surface soil of the northeastern Tibetan Plateau [4]. The average values of As, Cd, and Pb were higher than those of Qinghai Province and China as a whole (Table 2), while the average values of Cu and Ni were lower than the provincial and national values; all average Zn values were similar.

**Figure 2:**
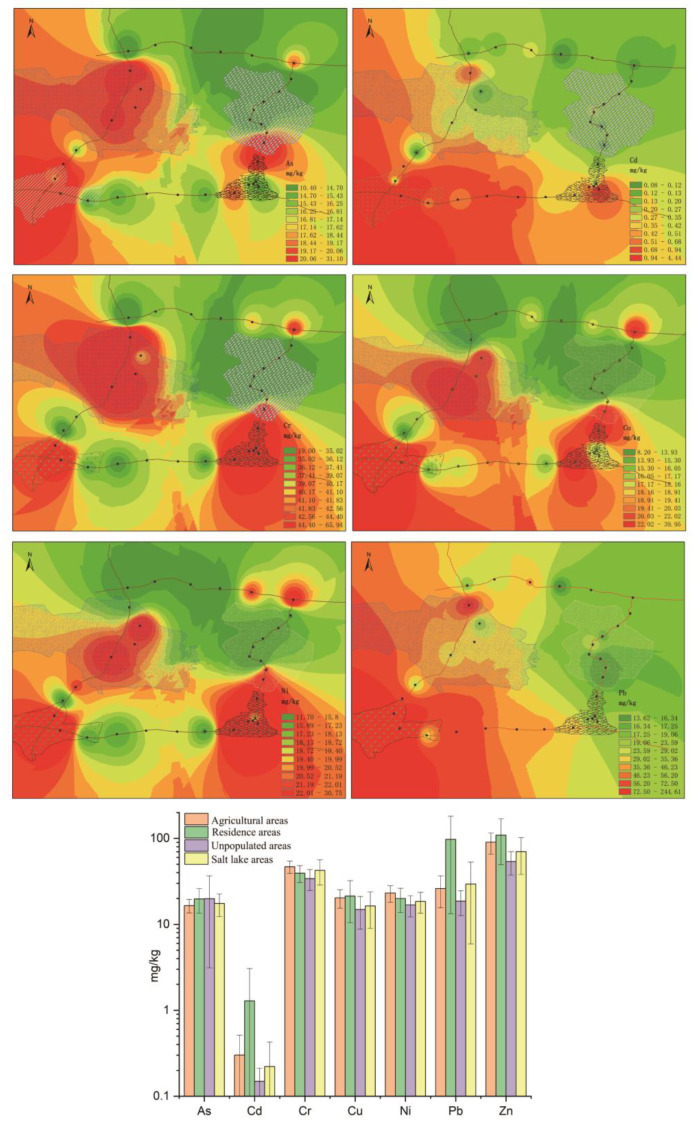
Spatial distributions of As, Cd, Cr, Cu, Ni, Pb, and Zn in agricultural areas, residential areas, unpopulated areas, and saline lake areas in the Qaidam Basin on the northeastern Qinghai–Tibet Plateau.

The spatial distributions of PTEs were obtained by deriving the spatial differences of the inverse distance weights of the PTEs (Figure 2). The highest values of As appeared in the unpopulated area (0816tr13), while the highest values of Cd, Cu, Pb, and Zn appeared in the residential area (0816tr01), which was located near a gas station. The maximum values of Cr and Ni were found in the saline lake area (0817tr05) and farmland area (0816tr12), respectively. By comparing the mean PTE values among different regions, we found few differences in the mean values of As, Cr, Cu, and Ni among different regions, the variations in the mean values of Cd, Pb, and Zn were relatively large. By combining these results with the spatial distributions of PTEs, the As, Cr, Cu, Pb, and Zn contents could be seen to be high in urban areas. However, the Cu and Ni element contents were high in agricultural areas.

### 3.2. Sources of PTEs in the Soil

Anthropogenic and diagenetic inputs are often mixed, and both contribute to the presence of PTEs in soils [10]. The correlation analysis results obtained for PTEs in different landscape regions are shown in Table 3. PCA was used to further confirm the sources of PTEs (Table 4). There was a significant correlation between Cd and Pb in unpopulated areas (R = 0.896; *p* < 0.01), and As, Cr, Cu, and Ni were well-correlated (R > 0.700; *p* < 0.01). This result was consistent with those obtained using PCA, and the high correlation indicates that these two groups of PTEs have different sources or have undergone different surface geochemical processes [14]. Previous studies have shown that the Pb and Cd contents measured in ice cores from the northeastern margin of the Qinghai–Tibet Plateau and the third pole are mainly of anthropogenic origin and were input through atmospheric deposition following large-scale transportation [37,38,39]. Unpopulated areas are similar to glaciers in that they are inaccessible and thus less affected by human activities. Therefore, Cd and Pb in unpopulated areas are significantly affected by pollutant input through atmospheric deposition on a long timescale. Previous studies have shown that the Cr in the surface soils of the northeastern margin of the Qinghai–Tibet Plateau is mainly generated by the natural weathering of the basin [11,15]. Therefore, the high correlations among As, Cu, Ni, and Cr and the high significance of the first principal component (PC1) indicate that the weathering of these elements is the main source of the PTEs in unpopulated watersheds.

In agricultural areas, the correlations among Pb, Cd, and Zn were good (R > 0.6; *p* < 0.05), and As, Cr, Cu, and Ni were also well-correlated (R > 0.6; *p* < 0.05). The PCA results showed that As, Cr, Cu, and Ni were significant factors in PC1 (R > 0.85) and that Pb, Cd, and Zn were significant factors in PC2 (R > 0.79). Previous studies have shown that large-scale planting increases the use of chemical fertilizers, thus leading to the enrichment of Cd and Zn in topsoil [26,40,41]. Moreover, Wang et al. [11] found that the Pb and Zn contents were relatively high in the farmland area of the Qinghai Lake basin in the northeastern part of the Qinghai–Tibet Plateau, with a good correlation. In agricultural areas, Pb and Cd were affected by farming activities. Therefore, the changes in the values of Pb, Cd, and Zn observed in agricultural areas in this study were closely related to human planting activities, while the As, Cr, Cu, and Ni contents were controlled by the regional soil background.

Previous studies have shown that the high Pb values in the surface soils of industrial and residential areas on the Qinghai–Tibet Plateau are mainly caused by vehicle exhaust emissions, industrial activities, and metal smelting [11,15,16,42]. In this study, high Pb values were distributed in the saline lake and Golmud City, where relatively frequent human activities occur. The accumulation of Pb in soils is intensified by the extensive exploitation of these saline lakes and smelting factories, so Pb is strongly influenced by human activities in these two regions. The correlations As, Cd, Cr, and Zn had with Pb were greater than 0.65, and their significances in PC1 were greater than 0.75, indicating that these PTEs were strongly influenced by human activities, while the correlation between Cu and Ni was 0.896, with a high significance in PC2. The Cu and Ni contents in the surface soils of the saline lake area were mainly consistent with natural background variations. In Golmud city, all PTEs had correlations greater than 0.740, and only one group arose in the PCA results; thus, in this region, Pb has been strongly influenced by human activities [4,15,22]. In addition, all PTEs analyzed in this study were strongly affected by human activities in urban areas; this region (Golmud city) is the region with the most intense human activity (and the highest population density) in the Qaidam Basin.

### 3.3. Variation of I_geo_, P_i_, NPI, and RI in the Soils of Study Areas

To judge the degree of PTE pollution more accurately in the study area, *I_geo_*, *P_i,_* and *NPI* were used in this study to discuss the sources and pollution degrees of PTEs. The inverse distance weight (IDW) method was used to obtain the spatial pollution degree distribution of the *p* results.

**Figure 3:**
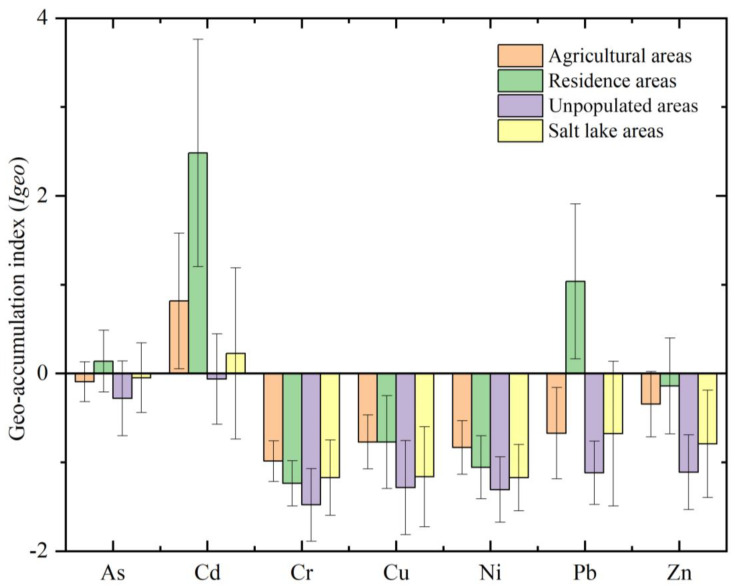
Average *I_geo_* values of PTEs in the agricultural areas, residential areas, unpopulated areas, and saline lake areas in the Qaidam Basin on the northeastern Qinghai–Tibet Plateau.

The *I_geo_* values of different PTEs were significantly different at different sites. The minimum and maximum *I_geo_* values were −2.26 (*I_geo_-Cr*) and 4.93 (*I_geo_-Cd*), respectively. The average *I_geo_* value for PTEs varied from −1.27 (*I_geo_-Cr*) to 0.484 (*I_geo_-Cd*). According to the classification criteria of *I_geo_* [32], all *I_geo_-Cr*, *I_geo_-Cu*, and *I_geo_-Ni* values in this study reflected similar changes, and more than 95% of the points where these metals were sampled were in the unpolluted category. *I_geo_-Zn* and *I_geo_-As* were in the unpolluted and unpolluted to moderately polluted categories, respectively, and the proportions of *I_geo_-Zn* and *I_geo_-As* in unpolluted sites were 87.5% and 68.8%, respectively. *I_geo_-Pb* was classified into four categories: unpolluted, unpolluted to moderately polluted, moderately polluted, and moderately polluted to heavily polluted, accounting for 81.25%, 12.5%, 3.13%, and 3.13% of the total sampling sites, respectively. However, Cd reached the heavily to the extremely polluted category, and the unpolluted, unpolluted to moderately polluted, moderately polluted, moderately polluted to heavily polluted, and heavy to extremely polluted categories accounted for 40.63%, 34.38%, 9.38%, 12.50%, and 3.13% of the total number of sampling points, respectively. The degree of PTE pollution was similar to that reported in previous studies [4,15]. The *I_geo_* results obtained for the analyzed PTEs were not consistent among different regions. According to the average *I_geo_* values derived in different landscape areas (Figure 3), the Cr, Cu, Ni, and Zn values in all landscape areas were less than 0, suggesting that Cr, Cu, Ni, and Zn are generally unpolluted in the study area. In residential areas, Cd is moderately polluted to heavily polluted, Pb is moderately polluted, and As is not polluted to moderately polluted, indicating that the PTE pollution in residential areas is the most serious. Human activities are the most frequent in residential areas among the four considered landscape areas, and the correlations among the PTEs and the PCA results also indicate that all PTEs in residential areas are affected by human activities. Golmud city is one of the most intense human activity areas in the Qaidam Basin and an important transportation hub on the Qinghai–Tibet Plateau. Previous studies have shown that railway and highway transportation processes will increase the enrichment of Cd and Pb in soils along the Qinghai–Tibet Plateau [21,43], so the Cd and Pb content in this region is strongly influenced by human activities. According to the classification criteria of *I_geo_*, the Cd contents in agricultural areas and saline lakes can be classified as unpolluted to moderately polluted.

The *P_i_* results are shown in Table 5. The minimum *P_i_* value was 0.31 (Cr), the maximum *P_i_* value was 45.88 (Cd), and the mean *P_i_* value varied from 0.65 (Cr) to 3.91 (Cd). The average *P_i_* values are ranked in the following order: Cd > As > Pb > Zn > Cu > Cr > Ni. According to the classification criteria of *P_i_* [33], more than 90% of the *P_i_* values of Cr, Ni, and Ni were distributed in nonpolluted areas, while the other values were distributed in mildly polluted areas. As was mainly slightly polluted (85.7%) and moderately polluted (11.43%). Zn was severe to moderately contaminated (2.86%) but was mainly found in nonpolluted areas (60.0%). Cd and Pb reached the level of severe pollution, with severe pollution index distributions of 25.71% and 5.71%, respectively; these two elements occurred as mild pollution and moderate pollution in 45.7% and 17.1%, and in 20% and 8.57% of the areas, respectively. Overall, the results obtained by P*_i_* were similar to those obtained for *I_geo_*, but the pollution level indicated by P_i_ was more serious than that indicated by *I_geo_*.

**Figure 4:**
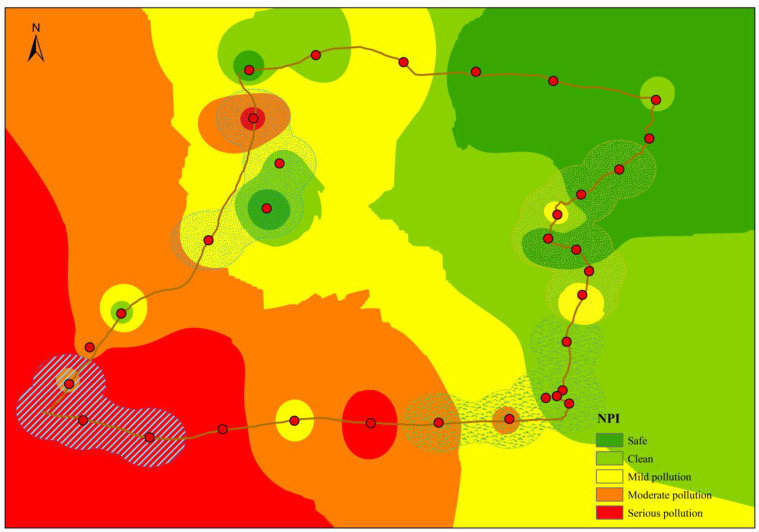
Spatial distribution of the *NPI* results obtained for the topsoils in the Qaidam.

The *NPI* results ranged from 0.56 to 32.56 (Table 5), with the highest value indicating intense pollution. The mean value was 5.93, indicating that seriously polluted areas are located in the study region. Combined with the pollution grade classification of *NPI* [34] and the spatial distribution characteristics of the pollution grade indicated by *P_i_* (Figure 4), the safe, clean, mildly polluted, moderately polluted, and severely polluted areas accounted for 18.4%, 20.8%, 23.4%, 18.9% and 18.5% of the total area, respectively. The safe areas mainly comprised the unpopulated area in the northeastern part of the research area, and clean areas were mainly distributed from unpopulated areas to farmland areas. Mildly polluted areas were mainly distributed in most of the saline lake areas and the farmland areas near Zongjia town in the south. Severe pollution was distributed in residential areas (e.g., Golmud city), while moderate pollution was distributed in the saline lake areas and the transition areas from farmlands to residential areas. Overall, the pollution levels in the study area showed an increasing trend from the northeast to the southwest. The pollution level distribution agreed well with the intensity distribution of human activities.

All pollution indices indicated that PTE pollution exists in some areas of the study area, requiring effective pollution control and remediation. According to the survey results, residential areas are the most polluted areas, and there is a phenomenon of pollution spreading outwards. Industrial and agricultural activities should also be carefully planned and managed to prevent possible pollution. This study provided comprehensive information regarding the distribution and pollution of PTEs in soils on the northeastern part of the Qinghai–Tibet Plateau and provided a basis for the formulation of effective environmental protection measures.

**Figure 5:**
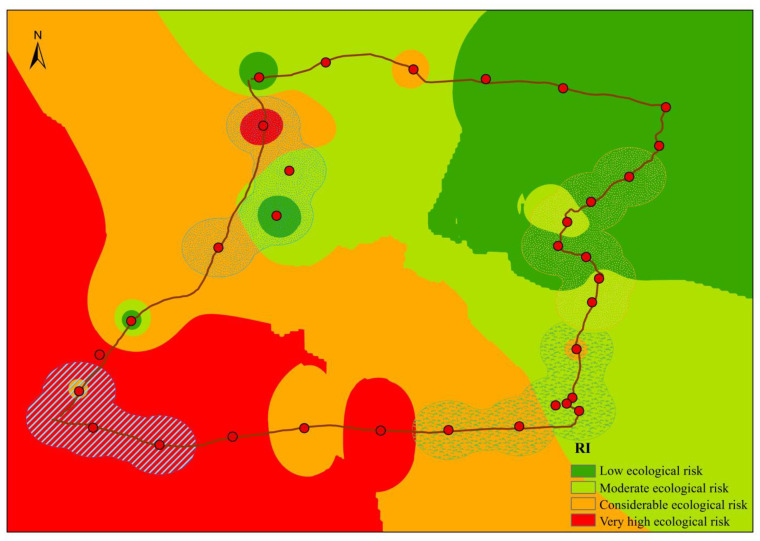
Spatial distribution of the *RI* results obtained for top soils in the Qaidam Basin on the northeastern Qinghai–Tibet Plateau.

The *RI* of soil PTEs in the study area varied from 8.3 to 216.4 (Figure 5). According to the ecological risk grade classification of the *RI* [33], there are 6 very strong ecological risk points, 6 strong ecological risk points, 9 medium ecological risk points, and 11 low ecological risk points in the region. Except for sampling points 0817TR03 and 0816TR13, the contribution of Cd to the *RI* of the other sampling points exceeded 50%, accounting for 51.2% to 93.7% of the total value. Cd controlled the changes in ecological risk coefficients at these points. The 0817TR03 and 0816TR13 points were controlled by As and Cd (77.8% and 73.6%, respectively); these points were located in low ecological risk areas. The spatial distribution of the *RI* results showed that areas with very strong ecological risks were mainly distributed in Golmud City, while the farmland areas and saline lake areas had strong to moderate ecological risks. The unpopulated areas mainly had low ecological risk.

Overall, the spatial distributions of PTE pollution and the potential ecological risk indices showed similar changes; both showed an increasing trend from the unpopulated areas in the northeastern Qaidam Basin to Golmud City in the south-western Qaidam Basin. The areas with serious pollution and very strong ecological risk were concentrated in Golmud City and spread to the surrounding areas. In addition, the six regions with high PTEs contents were mainly concentrated in this area, indicating that human activities, such as urban household waste discharge, transportation, industry, fuel production, and consumption, have become important sources of PTEs in soils along with the rapid development of the economy and urbanization in Qaidam Basin and the increase in soil PTE contents. Human activities have negatively impacted the soil’s ecological environment, so the ecological environments of soils in arid areas should not be ignored, especially regarding the PTE pollution of soils.

## 4. Conclusions

The mean values of PTEs in the soil of the study areas ranged from 0.02 (Cd) to 75.8 (Zn) mg/kg. The average values of As, Cd, and Pb were higher than those of Qinghai Province and China. The results of the correlation analysis and PCA showed that Pb and Cd were strongly affected by anthropogenic activites through large-scale transportation and regional input. Four methods were used to evaluate the pollution of PTEs in the soils of the study area. Cadmium posed the most serious contaminant in the study area based on *I_geo_* evaluation, with moderately polluted, moderately polluted to heavily polluted, and heavy to extremely polluted categories accounting for 9.38%, 12.50%, and 3.13% of the total number of sampling points, respectively. Cadmium and Pb reached the level of severe pollution, with severe pollution index distributions of 25.71% and 5.71%, respectively; these two PTEs occurred as mild pollution and moderate pollution in 45.7% and 17.1% and in 20% and 8.57% of the areas, respectively. *NPI* results illustrated that moderately polluted and severely polluted areas accounted for 18.9% and 18.5% of the total area, respectively. The potential ecological risk value ranged from 8.3 to 216.4. Golmud City and its surroundings were the most severely polluted areas in the region, accounting for 18.5% of the total area, which contained very high ecological risk. The spatial variations of PTE pollution and the potential ecological risk index were similar. They both increased from the unpopulated area in the northeastern Qaidam Basin to Golmud City in the south-western Qaidam Basin. The results indicate that human activities have negatively impacted the soil ecological environment in the rapid development of the economy and urbanization in the Qaidam Basin and these negative impacts tend to spread to unpopulated areas. Therefore, it is necessary to emphasize the significant impact of human activities on soil environmental quality and formulate preventive measures to reduce PTE pollution on the Qinghai–Tibet Plateau.

## Figures and Tables

**Table 1:** Classification of geological accumulation index (*I_geo_*), single factor pollution index (*P_i_*), Nemerow pollution index (*NPI*), and potential ecological risk index (*RI*).

*I_geo_*		*P_i_*		*NPI*		*RI*	
≤0	Unpolluted	≤1	Clean	≤0.7	Safe	<150	Low ecological risk
0–1	Unpolluted to moderately polluted	1–2	Mild pollution	0.7–1	Clean	150–300	Moderate ecological risk
1–2	Moderately polluted	2–3	Moderate pollution	1–2	Mild pollution	300–600	Considerable ecological risk
2–3	Moderately to heavily polluted	>3	Serious pollution	2–3	Moderate pollution	≥600	Very high ecological risk
3–4	Heavily polluted			>3	Serious pollution		
4–5	Heavily to extremely polluted						
≥5	Extremely polluted						

**Table 2:** Statistical summary of PTEs concentrations (mg/kg) in soil samples.

PTE	As	Cd	Cr	Cu	Ni	Pb	Zn
Minimum value	10.4	0.08	19	8.2	11.7	11.1	32
Maximum value	29.9	4.45	66	40	30.8	76.8	213
Mean value	18.2	0.38	39.57	17.3	19.1	34.5	75.77
SD	10.3	0.74	10.41	6.69	5.28	41.08	36.5
Qinghai Province *	14	0.137	70.1	22.2	29.6	20.9	80.3
China soil *	11.2	0.097	61	22.6	26.9	26	74.2

* Mean values of PTEs in the soil (data were given by [31]).

**Table 3:** Fitted linear relationship coefficients between each PTE in the four analyzed landscape types in the Qaidam Basin.

	Unpopulated Areas						
	As	Cd	Cr	Cu	Ni	Pb	Zn
As	1	−0.229	0.733 **	0.863 **	0.772 **	−0.260	0.498
Cd		1	−0.063	−0.093	−0.253	0.896 **	0.553
Cr			1	0.903 **	0.903 **	0.060	0.712 **
Cu				1	0.912 **	0.031	0.752 **
Ni					1	−0.195	0.578 *
Pb						1	0.638 *
Zn							1
	Agricultural areas						
	As	Cd	Cr	Cu	Ni	Pb	Zn
As	1	0.105	0.619	0.864 **	0.708 *	0.057	0.670
Cd		1	−0.410	0.029	−0.322	0.878 **	0.718 *
Cr			1	0.829 *	0.973 **	−0.503	0.264
Cu				1	0.895 **	0.023	0.698
Ni					1	−0.399	0.358
Pb						1	0.648
Zn							1
	Saline lake areas						
	As	Cd	Cr	Cu	Ni	Pb	Zn
As	1	0.847 *	0.955 **	0.452	0.483	0.857 *	0.883 **
Cd		1	0.876 **	0.063	−0.026	0.968 **	0.742
Cr			1	0.471	0.425	0.837 *	0.942 **
Cu				1	0.896 **	−0.023	0.708
Ni					1	−0.019	0.571
Pb						1	0.658
Zn							1
	Residential areas						
	As	Cd	Cr	Cu	Ni	Pb	Zn
As	1	0.888 *	0.743	0.852	0.778	0.945 *	0.841
Cd		1	0.759	0.942 *	0.752	0.967 **	0.975 **
Cr			1	0.923 *	0.995 **	0.828	0.750
Cu				1	0.917 *	0.963 **	0.946 *
Ni					1	0.842	0.748
Pb						1	0.965 **
Zn							1

* *p* < 0.05. ** *p* < 0.01.

**Table 4:** Factor loadings of components and those obtained after matrix rotation.

Unpopulated Areas	Component Matrix		Rotated Component Matrix	
	PC1	PC2	PC1	PC2
As	0.859	−0.273	0.882	−0.185
Cd	−0.036	0.962	−0.132	0.954
Cr	0.948	−0.004	0.943	0.091
Cu	0.985	−0.035	0.984	0.065
Ni	0.931	−0.233	0.949	−0.139
Pb	0.048	0.981	−0.051	0.981
Zn	0.777	0.615	0.711	0.690
Agricultural areas	Component matrix		Rotated component matrix	
	PC1	PC2	PC1	PC2
As	0.870	0.237	0.872	0.232
Cd	−0.098	0.959	−0.092	0.959
Cr	0.905	−0.366	0.903	−0.372
Cu	0.978	0.157	0.979	0.151
Ni	0.950	−0.260	0.949	−0.266
Pb	−0.165	0.953	−0.159	0.954
Zn	0.591	0.795	0.596	0.791
Saline lake areas	Component matrix		Rotated component matrix	
	PC1	PC2	PC1	PC2
As	0.977	−0.063	0.894	0.398
Cd	0.849	−0.515	0.992	−0.062
Cr	0.986	−0.077	0.909	0.390
Cu	0.546	0.815	0.105	0.975
Ni	0.500	0.825	0.059	0.963
Pb	0.818	−0.555	0.982	−0.112
Zn	0.959	0.179	0.766	0.604
Residential areas	Component matrix			
	PC1			
As	0.915			
Cd	0.953			
Cr	0.906			
Cu	0.991			
Ni	0.911			
Pb	0.986			
Zn	0.944			

Extraction method: Principal component analysis; rotation method: Varimax with Kaiser normalization.

**Table 5:** *P_i_* results were obtained for soil PTEs.

PTEs	*P_i_*			Distribution of *P_i_* (%)			
	Minimum Value	Maximum Value	Average Value	Clean (Safe)	Mild Pollution	Moderate Pollution	Severe Pollution
As	0.93	2.79	1.45	2.86	85.71	11.43	-
Cd	0.82	45.88	3.91	11.43	45.71	17.14	25.71
Cr	0.31	1.08	0.65	97.14	2.86	-	-
Cu	0.36	1.77	0.77	82.86	17.14	-	-
Ni	0.43	1.14	0.71	91.43	8.57	-	-
Pb	0.43	3.13	1.06	65.71	20.00	8.57	5.71
Zn	0.43	2.87	1.02	60.00	37.14	2.86	-

## Data Availability

Not applicable.

## References

[1] Kabata-Pendias A., Pendias H. (1999). Biogeochemistry of trace elements. Pwn Warszawa.

[2] Cong Z., Kang S., Zhang Y., Li X. (2010). Atmospheric wet deposition of trace elements to central Tibetan Plateau. Appl. Geochem..

[3] Shao D., Zhan Y., Zhou W., Zhu L. (2016). Current status and temporal trend of heavy metals in farmland soil of the Yangtze River Delta Region: Field survey and meta-analysis. Environ. Pollut..

[4] Wu J., Lu J., Li L., Min X., Luo Y. (2018). Pollution, ecological-health risks, and sources of heavy metals in soil of the north-eastern Qinghai-Tibet Plateau. Chemosphere.

[5] Padoan E., Romè C., Ajmone-Marsan F. (2017). Bioaccessibility and size distribution of metals in road dust and roadside soils along a peri-urban transect. Sci. Total Environ..

[6] Jin Y., O’Connor D., Ok Y.S., Tsang D.C., Liu A., Hou D. (2019). Assessment of sources of heavy metals in soil and dust at children’s playgrounds in Beijing using GIS and multivariate statistical analysis. Environ. Int..

[7] Kowalska J., Mazurek R., Gąsiorek M., Setlak M., Zaleski T., Waroszewski J. (2016). Soil pollution indices conditioned by medieval metallurgical activity—A case study from Krakow (Poland). Environ. Pollut..

[8] Wan D., Mao X., Jin Z., Song L., Yang J., Yang H. (2019). Sedimentary biogeochemical record in Lake Gonghai: Implications for recent lake changes in relatively remote areas of China. Sci. Total Environ..

[9] Kang S., Huang J., Wang F., Zhang Q., Zhang Y., Li C., Wang L., Chen P., Sharma C.M., Li Q. (2016). Atmospheric Mercury Depositional Chronology Reconstructed from Lake Sediments and Ice Core in the Himalayas and Tibetan Plateau. Environ. Sci. Technol..

[10] Wei B., Yang L. (2010). A review of heavy metal contaminations in urban soils, urban road dusts and agricultural soils from China. Microchem. J..

[11] Wang P., Cao J.J., Han Y.M., Jin Z.D., Wu F., Zhang F. (2015). Elemental distribution in the topsoil of the Lake Qinghai catchment, NE Tibetan Plateau, and the implications for weathering in semi-arid areas. J. Geochem. Explor..

[12] Deng W., Li X., An Z., Yang L. (2016). The occurrence and sources of heavy metal contamination in peri-urban and smelting contaminated sites in Baoji, China. Environ. Monit. Assess..

[13] González-Guzmán R., Inguaggiato C., Brusca L., González-Acevedo Z.I., Bernard-Romero R. (2021). Assessment of potentially toxic elements (PTEs) sources on soils surrounding a fossil fuel power plant in a semi-arid/arid environment: A case study from the Sonoran Desert. Appl. Geochem..

[14] Wang Q., Sha Z., Wang J., Du J., Hu J., Ma Y. (2019). Historical changes in the major and trace elements in the sedimentary records of Lake Qinghai, Qinghai–Tibet Plateau: Implications for anthropogenic activities. Environ. Geochem. Health.

[15] Li L., Wu J., Lu J., Min X., Xu J., Yang L. (2018). Distribution, pollution, bioaccumulation, and ecological risks of trace elements in soils of the north-eastern Qinghai-Tibet Plateau. Ecotoxicol. Environ. Saf..

[16] Zhang Z., Zheng D., Xue Z., Wu H., Jiang M. (2019). Identification of anthropogenic contributions to heavy metals in wetland soils of the Karuola Glacier in the Qinghai-Tibetan Plateau. Ecol. Indic..

[17] Wang X., Luo J., Lin C.-J., Wang D., Yuan W. (2020). Elevated cadmium pollution since 1890s recorded by forest chronosequence in deglaciated region of Gongga, China. Environ. Pollut..

[18] Centurion V.B., Silva J.B., Duarte A.W.F., Rosa L.H., Oliveira V.M. (2022). Comparing resistome profiles from anthropogenically impacted and non-impacted areas of two South Shetland Islands–Maritime Antarctica. Environ. Pollut..

[19] Łokas E., Zaborska A., Sobota I., Gaca P., Milton J.A., Kocurek P., Cwanek A. (2019). Airborne radionuclides and heavy metals in high Arctic terrestrial environment as the indicators of sources and transfers of contamination. Cryosphere.

[20] Ji X., Cheng Y., Abakumov E., Zhang H., Han C., Tang R., Wu D., Xie X. (2021). Desorption kinetics of heavy metals in the gleyic layer of permafrost-affected soils in Arctic region assessed by geochemical fractionation and DGT/DIFS. CATENA.

[21] Zhang H., Wang Z., Zhang Y., Hu Z. (2012). The effects of the Qinghai–Tibet railway on heavy metals enrichment in soils. Sci. Total Environ..

[22] Zhang H., Zhang Y., Wang Z., Ding M. (2013). Heavy metal enrichment in the soil along the Delhi–Ulan section of the Qinghai–Tibet railway in China. Environ. Monit. Assess..

[23] Bu J., Sun Z., Zhou A., Xu Y., Ma R., Wei W., Liu M. (2016). Heavy Metals in Surface Soils in the Upper Reaches of the Heihe River, Northeastern Tibetan Plateau, China. Int. J. Environ. Res. Public Health.

[24] Wei P., Shao T., Wang R., Chen Z., Zhang Z., Xu Z., Zhu Y., Li D., Fu L., Wang F. (2020). A study on heavy metals in the surface soil of the region around the Qinghai Lake in Tibet Plateau: Pollution risk evaluation and pollution source analysis. Water.

[25] Bing H., Wu Y., Zhou J., Li R., Luo J., Yu D. (2016). Vegetation and Cold Trapping Modulating Elevation-dependent Distribution of Trace Metals in Soils of a High Mountain in Eastern Tibetan Plateau. Sci. Rep..

[26] Dai L., Wang L., Liang T., Zhang Y., Li J., Xiao J., Dong L., Zhang H. (2019). Geostatistical analyses and co-occurrence correlations of heavy metals distribution with various types of land use within a watershed in eastern Qinghai-Tibet Plateau, China. Sci. Total Environ..

[27] Wang W., Ji X., Abakumov E., Polyakov V., Li G., Wang D. (2022). Assessing Sources and Distribution of Heavy Metals in Environmental Media of the Tibetan Plateau: A Critical Review. Front. Environ. Sci..

[28] Guo Z., Xie Z., Li J., Tian J., Zeng X., Ma W. (2021). Resource potential of the Jurassic gas system, northern margin of the Qaidam Basin, northwestern China. AAPG Bull..

[29] Doyi I., Essumang D., Gbeddy G., Dampare S., Kumassah E., Saka D. (2018). Spatial distribution, accumulation and human health risk assessment of heavy metals in soil and groundwater of the Tano Basin, Ghana. Ecotoxicol. Environ. Saf..

[30] Kuklová M., Kukla J., Hniličková H., Hnilička F., Pivková I. (2022). Impact of Car Traffic on Metal Accumulation in Soils and Plants Growing Close to a Motorway (Eastern Slovakia). Toxics.

[31] Ministry of Environmental Protection of the People’s Pepubic of China (MEPC) (1990). Background Values of Soil Elements in China.

[32] Müller G. (1969). Index of geoaccumulation in sediments of the Rhine River. Geojournal.

[33] Hakanson L. (1980). An ecological risk index for aquatic pollution control. A sedimentological approach. Water Res..

[34] Huang Y., Chen Q., Deng M., Japenga J., Li T., Yang X., He Z. (2018). Heavy metal pollution and health risk assessment of agricultural soils in a typical peri-urban area in southeast China. J. Environ. Manag..

[35] Facchinelli A., Sacchi E., Mallen L. (2001). Multivariate statistical and GIS-based approach to identify heavy metal sources in soils. Environ. Pollut..

[36] Yang S., Luo Y., Li Q., Liu W., Chen Z., Liu L., Liu X. (2021). Comparisons of topsoil geochemical elements from Northwest China and eastern Tibetan Plateau identify the plateau interior as Tibetan dust source. Sci. Total Environ..

[37] Bing H., Wu Y., Zhou J., Ming L., Sun S., Li X. (2014). Atmospheric deposition of lead in remote high mountain of eastern Tibetan Plateau, China. Atmos. Environ..

[38] Beaudon E., Gabrielli P., Sierra-Hernández M.R., Wegner A., Thompson L.G. (2017). Central Tibetan Plateau atmospheric trace metals contamination: A 500-year record from the Puruogangri ice core. Sci. Total Environ..

[39] Li Y., Huang J., Li Z., Zheng K. (2020). Atmospheric pollution revealed by trace elements in recent snow from the central to the northern Tibetan Plateau. Environ. Pollut..

[40] Yuan Y., Zeng G., Liang J., Huang L., Hua S., Li F., Zhu Y., Wu H., Liu J., He X. (2015). Variation of water level in Dongting Lake over a 50-year period: Implications for the impacts of anthropogenic and climatic factors. J. Hydrol..

[41] Liang J., Feng C., Zeng G., Gao X., Zhong M., Li X., Li X., He X., Fang Y. (2017). Spatial distribution and source identification of heavy metals in surface soils in a typical coal mine city, Lianyuan, China. Environ. Pollut..

[42] Xiang J., Wang J., Chen L., Ling Z., Han J., Li Q., Wang Q. (2019). Distribution, Source Identification, and Assessment of Potentially Toxic Elements in the Sediment Core from the Estuarine Region of the Golmud River to the Qarhan Salt Lake, Qinghai, China. Minerals.

[43] Zhang H., Wang Z., Zhang Y., Ding M., Li L. (2015). Identification of traffic-related metals and the effects of different environments on their enrichment in roadside soils along the Qinghai–Tibet highway. Sci. Total Environ..

